# Topographical and Biomechanical Guidance of Electrospun Fibers for Biomedical Applications

**DOI:** 10.3390/polym12122896

**Published:** 2020-12-03

**Authors:** Sara Ferraris, Silvia Spriano, Alessandro Calogero Scalia, Andrea Cochis, Lia Rimondini, Iriczalli Cruz-Maya, Vincenzo Guarino, Alessio Varesano, Claudia Vineis

**Affiliations:** 1 Department of Applied Science and Technology, Politecnico di Torino, 10129 Torino, Italy; silvia.spriano@polito.it; 2 Department of Health Sciences, Center for Translational Research on Autoimmune and Allergic Diseases–CAAD, Università del Piemonte Orientale UPO, 28100 Novara, Italy; alessandroscalia96@gmail.com (A.C.S.); andrea.cochis@med.uniupo.it (A.C.); lia.rimondini@med.uniupo.it (L.R.); 3 Institute for Polymers, Composites and Biomaterials (IPCB), National Research Council of Italy, Mostra d’Oltremare, Pad. 20, V. le J.F. Kennedy 54, 80125 Napoli, Italy; cdiriczalli@gmail.com (I.C.-M.); vincenzo.guarino@cnr.it (V.G.); 4 Institute of Intelligent Industrial Technologies and Systems for Advanced Manufacturing (STIIMA), National Research Council of Italy (CNR), Corso Giuseppe Pella 16, 13900 Biella, Italy; alessio.varesano@stiima.cnr.it (A.V.); claudia.vineis@stiima.cnr.it (C.V.)

**Keywords:** electrospinning, implantable devices, topographical guidance, biochemical guidance, contact guidance, functionalization, oriented fibers, random fibers

## Abstract

Electrospinning is gaining increasing interest in the biomedical field as an eco-friendly and economic technique for production of random and oriented polymeric fibers. The aim of this review was to give an overview of electrospinning potentialities in the production of fibers for biomedical applications with a focus on the possibility to combine biomechanical and topographical stimuli. In fact, selection of the polymer and the eventual surface modification of the fibers allow selection of the proper chemical/biological signal to be administered to the cells. Moreover, a proper design of fiber orientation, dimension, and topography can give the opportunity to drive cell growth also from a spatial standpoint. At this purpose, the review contains a first introduction on potentialities of electrospinning for the obtainment of random and oriented fibers both with synthetic and natural polymers. The biological phenomena which can be guided and promoted by fibers composition and topography are in depth investigated and discussed in the second section of the paper. Finally, the recent strategies developed in the scientific community for the realization of electrospun fibers and for their surface modification for biomedical application are presented and discussed in the last section.

## 1. Topographical and Biochemical Guidance: Electrospinning Potentialities for Production of Fibers

### 1.1. Electrospinning Basic Principles

Electrospinning is a simple and versatile process to produce nanofibers of a polymer from its solutions. The process is effective using polymers soluble in a volatile solvent. Electrospinning uses a strong electrostatic field to generate an electrically driven jet stream from an electrically charged liquid drop of the polymer solution to a collector able to gather the resulting dry nanofibers. The solution is fed through a metal nozzle (e.g., needle) connected to a high voltage generator at a fixed and controlled feed rate. Usually, the collector is electrically grounded. Main process parameters are applied voltage, solution feed rate, tip-to-collector distance, polymer concentration, electrical conductivity of the solution, solvent volatility, electrospinning time, and environmental conditions. Process parameters influences the characteristics of the resulting nanofibers (e.g., fiber size and morphology, layer density, and thickness) [1].

Investigations on the effect of process parameters on electrospun fiber size and morphology have been reported in several reviews [2,3,4]. Factors that influence the diameter of the fibers are: polymer(s) concentration in the solution, feeding rate of the solution, nature of the solvent used, electrical conductivity of the solution, and applied voltage. Molecular mass of the polymer can affect mainly the morphology of the electrospun fibers, from beaded to beadless fibers, increasing the molecular mass [5]. Further increases in molecular mass can lead to the production of flat fibers instead of fibers with a round section.

**Figure 1:**
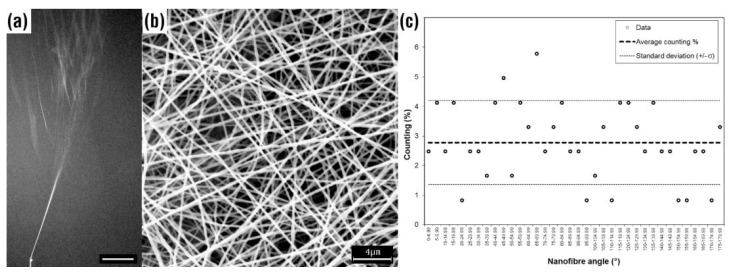
(**a**) Picture of the electrically driven jet produced by electrospinning from a charged metal tip (bottom) toward a collector (up). Scale bar: 2 cm. (**b**) Electron microscopy pictures of randomly oriented electrospun nanofibers collected on a static grounded flat metal collector (scale bar: 4 µm). (**c**) Plot of the nanofibers orientation (as result of 121 measurements on the nanofiber sample shown in (**b**), vertical 0°). The experiment was carried out using poly(ethylene oxide) (400 kDa) dissolved in water at 7 wt. % concentration and electrospun at 28 kV voltage, 1.8 mL/h flow rate, and 25 cm tip-to-collector distance. (Authors’ own pictures.).

As an example of electrospinning, Figure 1a shows the jet (direction is bottom-up) produced at 28 kV voltage from a metal tip fed with 1.8 mL/h of a water solution of 7 wt. % poly(ethylene oxide) (400 kDa average molecular mass). The jet of solution at the beginning linearly elongates toward a grounded metal collector (placed at 25 cm) because of the electrostatic repulsion force (Coulomb force) is maximum. As the electrostatic force decreases, the jet loses its straight path and starts to coil itself, while moving toward the collector (so-called whipping motion). In Figure 1a, the jet appears to break, at a given point, in a multitude of different jets, but it is just an illusion produced by the glints of the bending coils of the single jet, as excellently demonstrated in literature [6], as well as several electro-hydrodynamic models and theories predicting the path of an electrically driven jet in electrospinning [7,8,9]. In brief, an electrically-driven jets pushed from its source by repulsive forces undergoes to an axisymmetric motion accompanied by an axisymmetric stretching that reduce the jet radius and increase the length of the jet portion [4]. As the distance of electrical charges brought by the jet increases, the jet path becomes unstable by non-axisymmetric forces, so that a lateral motion of the jet is produced resulting in spiraling loops [9].

The solvent contained in the jet evaporates quickly and a dry fiber of the polymer is generated during the fly from the source to a collector. The fiber diameter is lower than the size of the liquid jet because of the solvent loss. Usually, the diameter of electrospun fibers ranges from few microns (microfibers) to sub-micron scale (nanofibers), and it is dependent on the nature of the polymer-solvent system and on the electrospinning parameters. The whipping motion of the jet path produces a randomly oriented fibrous structure (usually called non-woven) made up of a theoretically infinite long highly interconnected single fiber, as shown in Figure 1b. Based on several experimental observations and theories, electrospinning spontaneously produces an isotropic 2D structure, if external forces were not applied to the jet. Figure 1c reports the angles measured taking into account the bearing (from 0° to 179.99°) of the nanofibers with a vertical line as reference at a given point with the hypothesis of linear fiber development. To minimize errors, the measures were clustered every 5°. The average percentage counting over the resulting 36 clusters is 2.77% (dashed line). The standard deviation (σ) obtained from 121 measurements is 1.38%. The dot lines represent the 95% confidence interval. In agreement with the theoretical value, a near-perfect random orientation is obtained by electrospinning in this experiment.

Orientation of nanofibers can be attained to a certain extent applying additional forces to the fiber-forming fluid jet or to solid fibers by external devices or systems, as discussed in the Section 1.2.

As the electrostatic stretching of the fluid stream is not completed, because entanglements and viscoelastic forces are not enough to compensate electrostatic stresses, the morphology of electrospun materials changed from regular cylindrical fibers to small spheres (called beads) [10,11] and electrospinning turns into electrospraying.

On the other hand, viscous solutions associated with a rapid solvent evaporation can produce ribbon-like shaped nanofibers because of an uneven evaporation of the solvent along the jet cross section during the travel of the jet [12]. In that case, the skin of the jet quickly solidifies, while the bulk is still fluid, when the fiber lands on the collector; then, the solvent evaporates, and the fiber collapses, producing flat/bean-shaped cross sections [13]. On the contrary, solutions with a low viscosity and a high conductivity favors the formation of secondary jets from the main-stream that generates branching, which result in ultra-thin nanofibers [8,12].

**Figure 2:**
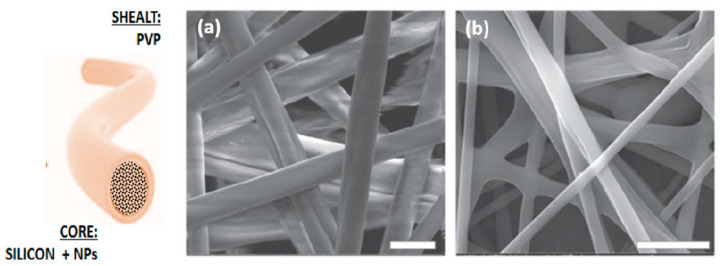
PDMS (Polydimethylsiloxane)/PVP core–shell fibers: SEM images of fibers (**a**) before and (**b**) after treatment in aqueous solution to remove the PVP shell (Scale bar 10 μm). Reproduced by V. Guarino et al., 2018, Mater. Res. Express 5 085029 [16].

Besides, an accurate control of the viscosity allows to combine different solutions by the implementation of more complex process configurations, i.e., based on use of co-axial needles, with promising results in a variety of applications. In the easier case, two solutions can be ejected independently through a coaxial capillary, a smaller inner capillary that fits concentrically into a bigger outer one, to form bicomponent fibers with core shell architecture. In this case, an accurate definition of the relative viscosities plays a crucial role in determining the uniform formation of core-shell jets and, ultimately, the morphology of the produced fibers. Generally, the core needs the use of more viscous solutions, while the use of lower viscosities is preferred for sheath where the flow is characterized by higher local tension areas [14]. This approach can be interesting for different uses. First, non-electric field sensitive solutions can be processed, in the core, only using good electrospinnable solution as external carrier. For instance, silicon nanofibers usually cannot be processed via electrospinning at room temperature for the relevant problems to dissolve the polymer in organic solvents [15]. In this case, the use of a coaxial configuration to fabricate tailor made core shell fibers that are efficacious to preserve the stability of the fiber core during the process. For instance, Polyvinylpyrrolidone (PVP) shell plays a protective role while the crosslinking of the inner core is going on, whereas it can be removed as the silicone core becomes stable (Figure 2) [16].

Highly soluble and/or degradable solutions can be variously use in the inner/outer counterpart to design a wide range of systems for the encapsulation and a time-controlled release of biological units—that is, cells, DNA fragments, genes—for the development of biologically active scaffolds [17]. Highly instructive biopolymers, i.e., proteins [18] and/or polysaccharides [19], can be used as sheath in combination with stable polymers, i.e., polyesters, polyurethanes, to support biological activities in vitro and in vivo.

### 1.2. Fiber Alignment

In the last twenty years, electrospun fibers have been variously investigated as three-dimensional scaffolds to improve the cell adhesion and proliferation [20,21]. To control the fiber alignment, flat collectors that are typically used in the basic setup configuration, can be replaced by rotating collectors (i.e., mandrels, wheels) that allow to preferentially impart fiber orientation by finely controlling rotation rates, from tens to thousands rpm [22,23]. Alternative methodologies to achieve aligned fibers concerns the use of “gap” collectors, which consist in the use of two conductive electrodes placed in parallel at a predefined distance, i.e., a gap from hundreds of micrometers to several centimeters, wherein local electric forces are able to arrange the charged fibers along preferential ways until to span the gap [23,24]. In the case of composite nanofibers embedded with magnetic particles, gap can be alternatively formed by the use of two permanent magnets that allow guiding the deposition of fibers along preferential ways by the application of magnetic forces on magnetically susceptive nanoparticles [25].

**Figure 3:**
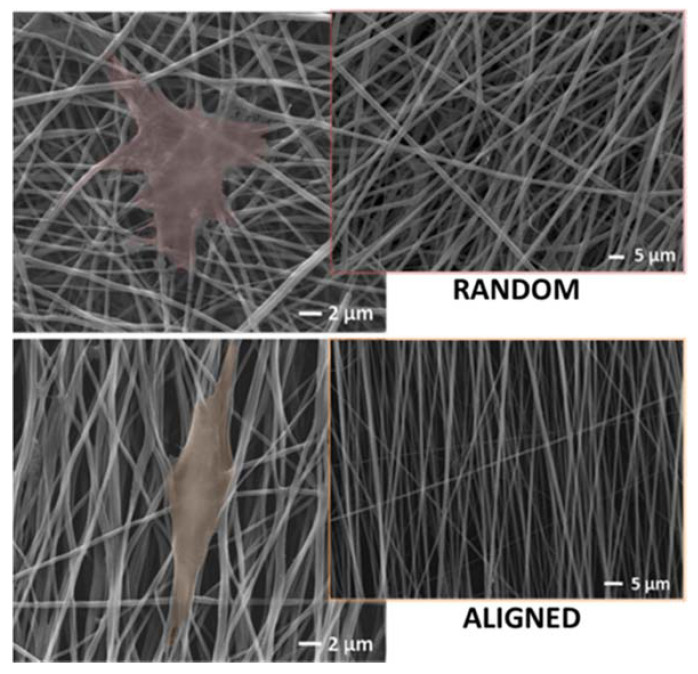
Bicomponent electrospun fibers fabricated by the combination of poly-ε-caprolactone and gelatin with random or aligned fibers organization (see into the square). Morphology of hMSC cell bodies influenced by the topological signals of the surrounding substrates. Image are adapted from V. Cirillo et al., J Mater Sci, Mater Med, 2014 [26].

Recently, it has been demonstrated that topographical features of electrospun fibers play an active role on regulating cell activities, including spreading, orientation, proliferation, migration, and differentiation of cells (Figure 3). Cirillo et al. investigated the morphology of adhered Human Mesenchymal Stem Cells (hMSC) bodies as a function of the surrounding pattern of fibers, remarking an effect of topographic signals on cell-to cell interactions and cytoskeleton re-modulation [26]. Other studies indicated that hMSCs were able to preferentially differentiate in the presence of aligned fiber patterns, with effects on cell-cell communication through gap junctions [27]. 

More recently, the effect of nanofibers alignment was investigated in in vitro cultures of differentiated cells cardiomyocytes, fibroblasts and cortical neurons [28,29,30]. In terms of cytotoxicity and cell proliferation, it was remarked all phenotypes exhibited good cell attachment, spreading and cell body alignment along the fiber’s axes. In particular, the preferential orientation of fibers promoted peculiar evidence as a function of the different phenotypes, i.e., the striation in the case of cardiomyocytes, branching in the case of cortical neurons. 

Electrospun scaffolds with uniaxial fiber alignment have been largely used to fabricate innovative scaffolds for peripheral nerve injuries, overcoming the well-known limitations of autologous or allogenic grafts treatments ascribable to different reasons (i.e., limited sources of autologous nerve, a second surgical site, and donor site morbidity, high rate of rejection) [31]. Contrariwise, aligned nanofibers show improved mechanical properties along preferential directions, giving the unique opportunity to form multilayered conduits with tubular architecture, suitable to assist nerve regeneration [32,33,34]. In this case, aligned fibers were preferentially arranged in the inner layer of the nerve guide conduit (NGC) in order to directly influence cell proliferation and migration, that are the driving mechanisms for peripheral neural regeneration. 

A key aspect concerns cell interaction of cells with aligned fibers and the contribution of mechanical properties of aligned fiber pattern on cell response in comparison with random fibers [35]. Several studies have not surprisingly reported about the improvement of mechanical response (i.e., tensile, flexural) of aligned fibers with respect to random ones, due to the macroscopic (i.e., higher physical interactions and fiber packing) and microscopic (i.e., higher crystallinity) properties induced by the basic principle of electrospinning process. Aligned fibers can be variously assembled to form anisotropic structures with heterogeneous tensile properties along different ways, i.e., differences of over one order of magnitude in the elastic modulus from 0° to 90° orientation angles with respect to the applied load direction, thus providing a selective transfer of biomechanical stimuli to cells as required for the repair of tissues as tendon, nerve, skin, and bone, as well as cartilage [35,36,37]. It is well known that the actin cytoskeleton of cells follows the aligned pattern of fibers and helps cells to recover from stretch during mechanical loading [38]. Moreover, the biomechanical cues also allow for the maintenance of cell phenotype without alteration of cell function [39], also influencing the cell differentiation of mesenchymal stem cells through mechanotransduction pathways [40]. 

Additionally, the high versatility of the electrospinning technology even offers the possibility to collect aligned fibers to impart a preferential patterning to the device surfaces [41]. Recently, Ferraris et al. investigated the use of aligned fibers to impart topographical cues to the surface of titanium plugs, thus improving the interface between materials and surrounding cells [42]. In this case, they demonstrated that aligned electrospun fibers deposited onto titanium surfaces concur to guide fibroblast growth along fibers, thus promoting the healing of soft tissue around transmucosal implants. Recent results also confirmed that fibroblasts grew along the submicrometric fibers; thus, this strategy can reduce the failure of transmucosal implants due to the lack of adhesion of soft tissues [43].

Lastly, aligned electrospun fibers can be successfully used for drug delivery, due to the strict relationship between morphological features (i.e., high-surface-to-volume ratio of fibers) and controlled release mechanisms of molecules and drugs [44]. Generally, both release profiles of random and aligned fibers, characterized by an initial burst release followed by a sustained release of the molecule. However, there are some differences. In terms of release, kinetics and drug encapsulation can be detected as a function of the fiber organization and anisotropy. For instance, Eslamian et al. verified that an initial burst release of anti-inflammatory drugs (i.e., dexamethasone) tends to be more pronounced in the case of random fibers rather than aligned fibers, due to the higher probability to form surface pores forming as slowing evaporation mechanisms of the solvent occur [45]. Accordingly, aligned fibers showed a lesser burst and a more sustained release of the drug compared to the random fibers, mainly due to a different degradation behavior, thus suggesting their promising use for tissue regeneration and nanomedicine [46,47].

### 1.3. Natural vs. Synthetic Fibers

**Figure 4:**
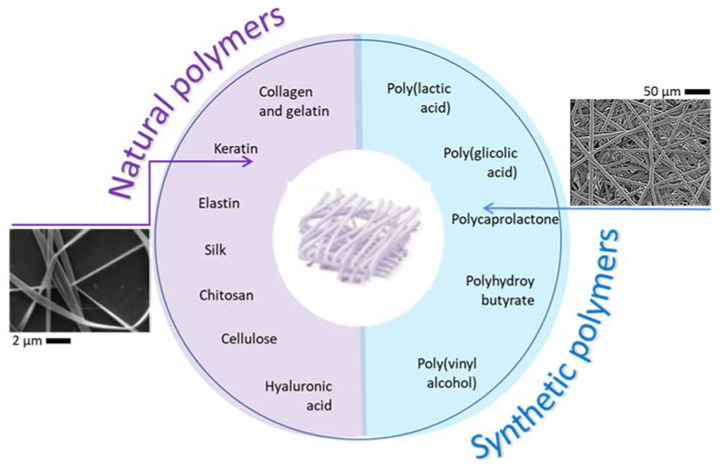
Synthetic and natural polymers used for the fabrication of electrospun fibers. On the left, Keratin nanofibers with sub micrometric diameters (reproduced by Cruz-Maya et al. [53]). On the right, poly-ε-caprolactone (PCL) microfibers with micrometric fiber diameters (reproduced from Saracino et al., Mater Sci Eng, 2021 [39]).

The manipulation of material chemistry and processing technologies allows improving structural and functional properties of tailor-made devices by the assembly of various materials combination or the ex novo synthesis of new materials with new biochemical functionalities to design innovative biomaterials for tissue engineering [48]. For instance, the manipulation of different materials may actively contribute to the design of highly complex platforms able to reproduce interface tissues, such as meniscus, cartilage, and intervertebral disc [49,50,51], as well as requiring the re-invention/adaptation of conventional processing techniques for the fabrication of multilayered systems with chemical/physical gradients and multiple functionalities. Hence, it is mandatory to begin from a solid knowledge about materials properties to properly design tailored scaffolds for optimizing, case by case, repair and regeneration strategies. More generally, an ideal biomaterial should be biocompatible and bio-adhesive, possess adequate mechanical properties to tolerate the applied physiological load and, finally, show good bioactivity to assure an efficient bonding at the material/tissue interface. The criteria for selecting the biomaterials may be based on their chemistry, molecular weight, solubility, shape and structure, hydrophilicity/hydrophobicity, lubricity, surface energy, water absorption, and degradation properties [52]. Accordingly, polymers commonly used for the electrospinning can be distinguished into natural or synthetic ones, as a function of their specific peculiarities (Figure 4).

In particular, synthetic polymers exhibit better mechanical properties than natural polymer. Meanwhile, natural polymers are better recognized from a biological point of view. A large variety of electrospun fibers made of synthetic or natural polymers have been in vitro investigated [54]. However, the best results were univocally obtained in the case of bicomponent electrospun fibers formed by the combination of both synthetic and natural polymers to simultaneously reach desirable properties, in terms of mechanical strength and biocompatibility [18,55,56,57].

Synthetic polymers, such as biodegradable polyesters, including poly-ε-caprolactone (PCL), poly(lactic acid) (PLA), poly(glycolic acid) (PGA), poly(lactic-co-glycolic acid) (PLGA), and their copolymers, have been widely used to fabricate electrospun scaffolds for tissue engineering [58,59]. These polymers are U.S. Food and Drug Administration (FDA) approved, due to their good biocompatibility and biodegradability. Furthermore, their peculiar physico-chemical properties enable to dissolve them into a wide set of organic solvents (i.e., polar or apolar), thus giving the opportunity to variously process them to design different devices in terms of porous structures, sizes, orientation, shape, structures, and tailored degradation rate as a function of molecular weight, composition, and microscopic properties (i.e., crystallinity degree) [60,61,62,63]. 

As a function of their chemical structure and functionalities, synthetic polymers can present different interaction with cells in vitro. For instance, hydrophobic or partially hydrophobic polymers, such as PCL, PLA, PGA, and their copolymers, show different surface wettability with respect to hydrophilic polymers, such as PEG or PVA, that drastically alter the basic mechanisms of cell-material interaction [64,65]. In particular, an improvement in cell proliferation can be recognized in the presence of hydrophobic fibers only at the early stage, while cell proliferation significantly increases in the long period; in the case of more hydrophilic ones [66], which mainly promote the mechanisms of protein adsorption [67]. In this perspective, recent approaches involve the use of surface modification or post-treatments of synthetic-based materials [68,69]. For instance, PCL fibers have been treated after the electrospinning process with NaOH to improve their hydrophilicity without altering the fibers morphology and improving their biocompatibility [70,71].

For in vitro studies, electrospun fibers made of synthetic polymers, first of all, have to provide a structural support for cells during the regeneration process, basically assuring their chemical/physical stability until the new tissue has been synthetized [72]. In this context, microscopic, i.e., molecular weight, and macroscopic (i.e., crystallinity) of the fibers play a crucial role on the biomechanical and biochemical properties of the scaffolds that are mainly influenced by the polymer degradation [73,74]. For instance, aliphatic polyesters are the most widely used synthetic polymers due to their balanced behavior in terms of biocompatibility and biomechanical stability. In this case, polymer crystallinity may affect the permeability and degradation kinetics, thus influencing the ability of cells to adhere and proliferate [75]. However, this mechanism is not completely discovered, and it seems to be different as a function of the peculiar properties of the polymer solution used that can address different crystallinity degree into the electrospun fibers as a function of the polymer chemistry and composition [74,76]. Accordingly, Guarino et al. demonstrated that the use of highly polar solvents coupled with low concentration of the PCL solution tends to form more crystalline fibers rather than high concentrated solutions and low polar/apolar solvents. Other studies also suggested a further contribution of polymer crystallinity on cellular response mediated by changes in surface roughness at the nanometric scale [77,78], which is universally recognized to play a crucial role on the interface between cells and surrounding material.

Currently, electrospun nanofibers have found varied use, especially in biomedical and healthcare applications, above all thanks to their particular properties, such as high solubility, high surface-volume ratio, and having wide usage areas they have provided. Natural materials have been recently considered for biomedical applications with the aim of overcoming the limitations of synthetic materials. They have the advantage of containing, in their structure, sequences of signals that promote and maintain cell adhesion and functions. With the use of peptide-protein structures derived from plant and animal, it is possible to obtain biodegradable and biocompatible fibers. Collagen, keratin, fibroin, gelatin, and chitosan are the most used natural biomaterials of protein derivation and are proposed for tissue engineering [79]. Plant-derived proteins used in nanofiber production are zein, soy protein, and gluten, and animal derived proteins are keratin, fibroin, casein, hemoglobin, elastin, bovine serum albumin, collagen, and gelatin [80]. Electrospun nanofibers mimic the natural extracellular matrix (ECM) and the sustained release of biomolecules and drugs at the targeted site. Nanofibers are considered to be the most important nanostructured material that can be applied in the biomedical field, as drug delivery systems [81], as well as 3D constructs for tissue regeneration of skin [82], bone [83], cartilage, neural tissue [84], heart valves, and muscle. Scaffolds of electrospun collagen nanofibers, collagen hydrogels, and collagen microfiber scaffolds are some examples of formulations that can be used for skin regeneration. The electrospinning process was used to produce nanofibrous scaffolds as skin substitutes of collagen type I and type III that mimic the molecular and structural properties of native collagen, or to coat scaffolds made from other materials and increase their biocompatibility. Electrospinning is a rapid and efficient technique that can be utilized to produce complex, three-dimensional shapes and seamless scaffolds. Moreover, with the electrospinning process, it is possible to control the chemical composition and material properties of the engineered scaffolds. A further use of electrospinning can be to introduce subtle structural properties into an engineered material by adjusting the orientation of the fibrils within the fabricated mesh. Electrospinning collagen assists cell growth and cell penetration into the engineered matrix. The structural and biological properties of nanofibers electrospun collagen make it an excellent material for tissue engineering [85]. 

Gelatin is a partially hydrolyzed version of collagen and contains a high number of amino acids, such as glycine, proline, and 4-hydroxyproline. Gelatin nanofiber scaffolds have also shown possible applications in wound healing regeneration. Gelatin is better than collagen as it is less immunogenic and therefore allows for greater cell adhesion. Scaffolds made of gelatin nanofibers using the electrospinning method have shown potential applications in wound healing processes. Due to their low mechanical strength, collagen and gelatin cannot be used as scaffolds for hard tissue [86].

Keratins are animal structural proteins characterized by a high sulfur content. These proteins are present in different biomasses (such as wool, hairs, feathers, hooves, horns) with different typical molecular masses. Extraction methods uses mainly reductive or oxidative chemicals [87]. One of the most important features of keratins is the large amount of cysteine forming intra- or inter-chain disulfide-bonds. Keratins are versatile proteins for the production of flexible films, sponges, fibers, and nanofibers. In particular, pure keratin can be transformed into nanofibers by electrospinning from solutions of solvents, such as formic acid [88] and 1,1,1,3,3,3-hexafluoro-2-propanol [53]; on the contrary, keratin-based nanofibers have been obtained from water solutions by the addition of synthetic water-soluble polymers, like poly(ethyl oxide) [89], polyvinyl alcohol [90], polyvinylpyrrolidone [91], and polycaprolactone [53].

Keratin-based biomaterials have been investigated recently due to their excellent biocompatibility and intrinsic biological properties. In fact, keratins promote the adhesion of several kinds of cells and have been proposed for coating for the cell culture dishes and implantable devices [92,93]. Keratin is known for its ability to support and stimulate fibroblast [37,94,95] and osteoblast [96] cells growth. Moreover, keratin can bind metal ions, such as silver, to obtain antibacterial properties [97]. Finally, the stability of keratin-based biomaterials can be improved by thermal treatments [90,98,99] that induce the formation of cross-linking between molecular chains, making keratin insoluble in water and durable.

Another natural polymer used in tissue engineering is silk fibroin, a fibrous protein of silk core. Fibroin is an interesting biomaterial, and it can be exploited to produce scaffolds in the form of fibers, hydrogels, and sponges. There are two types of silk proteins: fibroin, which is hydrophobic; and sericin, which is hydrophilic. Fibroin, being biodegradable and biocompatible, provides great permeability for nutrients and has been used to design scaffolds for tissue engineering applications. Due to its high biocompatibility and minimal inflammatory reaction, fibroin is useful in skin grafts and wound dressings. Fibroin electrospun scaffolds, being porous and cytocompatible, have shown very promising in wound healing and regeneration of skin tissue [100]. 

Chitosan is a biomaterial of polysaccharide origin derived from a component of the exoskeleton of arthropods and crustacean shells, called chitin. Preparation of chitosan-based scaffolds is rather difficult due to its ionic characteristics, but, once produced, chitosan nanofibrous scaffolds give better adhesion, proliferation, and differentiation of keratinocytes, fibroblasts, and endothelial cells, as well as improved vascularization and granulation tissue formation, with respect to two-dimensional (2D) films and 3D chitosan sponges. Chitosan has numerous anti-microbial, hemostatic, and antifungal properties that make it suitable for a wide range of applications in wound and burn treatments [101]. For regenerative and wound healing applications in skin tissue engineering, hydrogels, such as hyaluronan-fibronectin hydrogel and chitosan-gelatin hydrogel, in combination with PLGA nanofibrose scaffold, were used. 

Zein is a water insoluble bio-macromolecule and prolamin rich plant protein. This protein is used because it provides bioactive and drug compounds and has a controlled release capacity and high bioavailability of the loaded compounds. Zein nanofibers have been used for the electro-encapsulation and stabilization of food bioactives, such as essential oils, aromas, and controlled release of active additives [102]. Heydari-Majda et al. produced zein nanofibers loaded with Barije essential oil that has anti-diabetic and antioxidant properties, useful in the production of new delivery vehicles for diabetes control [103]. 

### 1.4. Composite Nanofibers

Composite nanofibers are commonly composed of heterogeneous materials with peculiar properties mainly determined by the single components in terms of composition, structure, and interfacial properties. They are generally processed by the dispersion in solution of 1D materials, namely one dimension outside the nanoscale (i.e., nanotubes [104], nanorods [105], nanowires [106], nanoneedles [107]) or 2D materials, namely two dimensions outside the nanoscale (platelet or flesh-like structures, such as graphite [41] or/graphene oxide [108], nanofilms, or nanolayers. Firstly, an accurate control of the physical aggregation and/or 1D materials orientation allows influencing structural of the composite nanofibers, addressing their use for different application in the biomedical field. Secondly, their fine distribution at the interface with the polymer matrix strongly contribute to particularize interface properties in terms of surface energy as a function of their peculiar shape factors, with relevant effects on biomechanical response and stability. Lastly, intrinsic properties of nanophases (i.e., electric, magnetic, optical) concur to impart peculiar functional properties to composite nanofibers suitable for innovative applications in tissue regeneration, diagnostic/theranostic and biosensing applications. For instance, electrospun nanofibers based on polymer/carbon nanotube (CNT) composites are very attractive to design composite nanofibers that combine remarkable mechanical and electronic properties. In particular, the application of electrostatic forces contribute to a preferential alignment of CNTs within the nanofibrous structure that can greatly improves the electrical and mechanical properties of fibers with effects on in vitro cell response [109]. Graphene oxide nanophases were embedded into PCL and gelatin nanofibers to produce innovative scaffolds with superior properties in terms of hydrophilicity, biodegradation features, and electrical conductivity suitable for neural tissue engineering, as well as controlled drug delivery [110]. More recently, fluorescent nanodiamonds (fNDs) were integrated into electrospun poly-ε-caprolactone (PCL) fibers to form composite nanofibers suitable as innovative platforms for imaging and sensing in biology, due to their peculiar features in the optical detection of temperature and magnetic fields at the nanoscale [111].

## 2. Topographical and Biomechanical Guidance: The Biological Standpoint

### 2.1. Contact Guidance

The extracellular matrix (ECM) is a complex macromolecular network of proteins composed by collagens, elastin, fibronectin, laminins, and several other glycoproteins, depending on tissues, which comprise the acellular part of tissues and organs [112]. ECM has been classified into two major groups based on structure and composition: the interstitial matrix (ICM) surrounding cells, without direct contact, and the pericellular matrix (PCM), in close contact with the cells. The difference between them is strictly related to the proteoglycans: PCM proteoglycans are mainly represented by perlecan, agrin, and collagen type XV and XXVIII, while ICM proteoglycans mostly diversify depending on different tissue [112].

**Figure 5:**
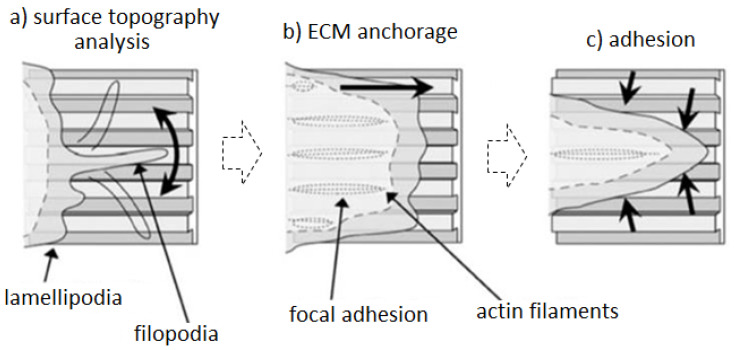
Contact guidance machinery. Lamellipodia extrusions are temporarily exposed following filopodia sensors information (**a**) prior to the surface anchorage by focal adhesions (**b**) and full adhesion by cytoskeleton rearrangement (**c**). (Authors’ own pictures.).

The cell-matrix interaction machinery starts with the formation of the focal contacts [113], representing the “anchors” used by cells to adhere. Accordingly, once the sensors completed their surface analysis, a regulatory signal is transmitted from the cytoskeleton to the nucleus to optimize cells’ adaptation to the surface, thus enabling actin filaments spread and ECM deposition. This phenomenon is known as contact guidance to describe the cells’ ability to adapt and move along oriented tissue or surfaces [114]; it has been observed during tissue regeneration, wound healing, and cancer progression [115], and it is exploited in tissue engineering for the surface modification of implantable bio-instructive biomaterials. The cell capability to respond to micro- and nano-topographies is due to the sensors lamellipodia and filopodia synchronous activity, as schematized in Figure 5 [114]. 

In fact, lamellipodia are temporary extensions of the cytoskeleton responding to the sensory and exploratory activity exploited by the filopodia [116]. So, the cell’s movement starts with the filopodia attachment to ECM collagen fibers via integrins and continue when the cell cytoskeleton contraction breaks the cell-matrix anchorage, thus allowing the cell moving forward [117]. Although filopodia are important, lamellipodia play the pivotal role for the surface topography analysis; for example, Ballestrem et al. [118] demonstrated that the inhibition of the lamellipodia activity makes the cells unable to adapt to surface modifications, thus lacking a proper adhesion. The contact guidance mechanisms are regulated by different factors related to the substrate, such as the fibers orientation and distribution, or to the cells’ metabolism. Agudelo-Garcia et al. [119] demonstrated that oriented matrix fibers strongly increase cell motility in comparison to random fibers, while Pourfarhangi et al. [120] showed that contact guidance can be cell-cycle dependent, too, because cells in G1-phase exhibited higher migration parameters than cells in G2- or S-phase. Ramirez-San Juan et al. [121] demonstrated how fibers spatial distribution regulates contact guidance; in particular, they showed that a distance ≥5 µm between aligned fibers represent the best condition to improve the guidance due to the forced lamellipodia alignment towards oriented ECM. 

### 2.2. Biomechanical Guidance

In the past, ECM has been considered only as a natural substrate responsible for the physical integrity of the tissue; today, new evidence has highlighted an active role of the ECM regulating many cellular processes including growth, migration, differentiation, and homeostasis [122]. Of course, all of these ECM-driven processes require regulatory pathways involving direct connections with the cells that are mediated by several cell surface molecules, including different integrins, proteoglycans, receptors (e.g., Discoidin domain receptor (DDRs) and Robo), syndecans, and Cluster of differentiation 44 (CD44) [123]. Among them, integrins have been recognized to play the pivotal role within the regulatory cascade. They are defined as transmembrane heterodimer molecules interacting with the matrix by their cytoplasmatic tail [123]. Their activation can be regulated by two different mechanisms: the ‘’outside-in’’ mechanism involving cell-ECM direct interaction and the ’inside-out’’ machinery activated by extracellular events [123]. In detail, when the actin filaments from the cells’ cytoskeleton bind to the ECM integrins, a talin-dependent increase in adhesion strength occurs. Therefore, a signal is generated with the activation of the GTPase and GTP binding protein Ras-related C3 botulinum toxin substrate 1 (Rac) and Cell division control protein 42 homolog (Cdc42), which induce the formation of lamellipodia and filopodia, respectively. The selective binding between integrins and ECM proteins requires a broad class of the former: so, 24 different integrin heterodimers formed by 18 alpha subunits and 8 beta subunits can be distinguished [123]. Table 1 summarizes the ECM proteins (substrates) related to the specific integrins subunits. 

Among all the subunits, β_1_ is considered as crucial due to its connection with several α subunits and key proteins, such as filamin [124]. Moreover, the subunit β_1_ is fundamental for cytoskeleton-collagen I interaction; for example, Zhang et al. [125] demonstrated that after β_1_-integrin knockdown, tumorigenic MDA-MB-231 cells drastically decrease their migration and spread ability.

Other crucial integrins are α_6_β_4_, αLβ_2_, αMβ_2_, αXβ_2_, and α_4_β_1_. The first one (α_6_β_4_) is expressed in the hemidesmosome during epithelial tissues and ECM interaction; if silenced, a drastic decrease of epithelial cell adhesion to the connective tissue can be observed [126]. The other three (αLβ_2_, αMβ_2_, αXβ_2_) bind the protein ICAM-1, which is very important in stabilizing cell-to-cell interactions. The last one (α_4_β_1_) regulates the immune cells adhesion to the vascular endothelium. Two extra integrins, α_V_β_3_ and α_V_β_5_, are involved in coordination and movement of several cell types interacting with vitronectin, fibronectin, and collagen IV. 

The emerging literature suggests that all those integrins are not passive molecules, but their expression is due to a regulatory machinery related to the ECM rigidity. As a clarifier example, integrins α_5_β_1_ and αvβ_6_ bind the same substrate (fibronectin) but exhibit different rigidity thresholds, thus forming mature or immature adhesions supporting different tension forces [127].

Recently, it has been demonstrated that integrins are not only involved in the cells-ECM adhesion regulation, but they can also be considered as crucial regulators of several other cellular processes. In fact, the pathways regulated by integrins are sometimes very similar to those ones mediated by growth factors and cytokines. For example, integrins can be the precursors of the SFK-Fak-p130Cas signaling by the subunit α_v_β_3_, thus influencing many other cascades (such as Phosphoinositide 3-kinase (PI3K), Protein kinase C (PKC), and Ras) related to cell survival, control of transcription, migration/invasion, proliferation, cell motility, and cytoskeletal organization [128].

**Figure 6:**
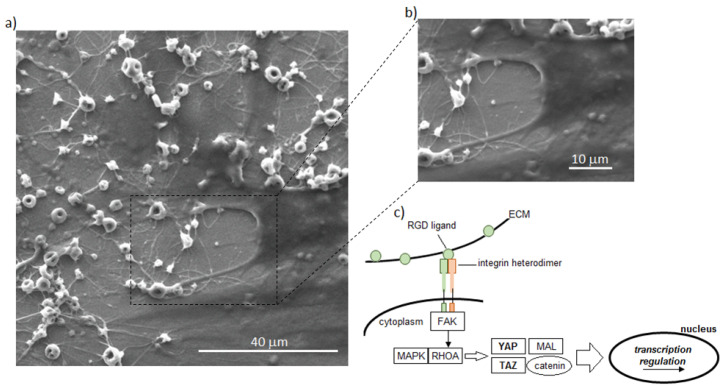
Biomechanical guidance. Keratin nanogrooves and nanofibers were used to drive cells adhesion and spread exploiting their biomechanical guidance towards cytoskeleton (**a**,**b**). This process involves the activation of different pathways mainly related to the Yes-associated protein (YAP)/TAZ nuclear signaling (**c**). (Authors’ own pictures).

The reason why cytoskeleton adaptation to the ECM proteins via integrins is sensed by the nucleus is that the latter are linked to mechanosensing molecules, such as Yes-associated protein (YAP) and the transcriptional coactivator with PDZ-motif (TAZ). YAP/TAZ act synergistically with components of the Hippo pathway (RhoA and RhoA-effector proteins), thus affecting the cytoskeleton tension. YAP and TAZ activation depend on their accumulation into the nucleus, and this, according to several studies [129,130,131,132,133], is under the control of stimuli, like cell shape, ECM stiffness, and shear stress, as schematized in Figure 6c, by exploiting keratin nanogrooves and nanofibers as biomechanical attractors. In fact, it has been demonstrated that YAP and TAZ are localized in the cytoplasm of cells experiencing low level mechanical stress, such as cells attached to a soft hydrogel matrix (0.7–1 kPa), while they migrate inside the nuclei when cells are attached to rigid substrates, such as polystyrene (15–40 kPa), or during deformation or cytoskeletal tension. 

The fact that YAP and TAZ requires an intact cytoskeleton is demonstrated by several in vitro experiments [134], even if the exact mechanism of how cytoskeleton have a role in YAP and TAZ signaling in the context of mechanotransduction is not completely understood. What is already stated is that once a mechanical stress is sensed by the cell trough integrin-ECM interaction, the signal must be propagated as fast as possible; this is mediated by mechanosensory proteins, such as vinculin and other focal adhesion kinases, like Src-family kinases that connect cytoskeletal proteins to the ECM.

Similarly, other surface molecules, such as DDRs, CD44, and Robo, are involved in cell growth, migration and differentiation. The Discoidin domain receptors (DDRs) are tyrosine kinase receptors recognizing different collagens isoforms; their ligands are divided into two different groups named DDR1 and DDR2. DDR1 is composed by 5 different isoforms (1a,1b,1c,1d,1e), while DDR2 is composed by only one protein [135]. In particular, Valiathan et al. [136] demonstrated that DDRs function is strongly related to cells’ differentiation fate; accordingly, DDR1 was found promoting murine axon formation, osteogenic differentiation in human mesenchymal stem cell, epithelial differentiation, and murine myoblast differentiation, while DDR2 promoted differentiation of murine osteoblast and chondrocytes, suppressing murine differentiation of pre-adipocytes to functional adipocytes [137].

CD44 is a member of the cell adhesion molecules (CAMs) that have a large role in cellular adhesion and signaling for cell growth, proliferation and motility. This ubiquitous molecule was found holding different isoforms interacting with both normal and cancerous cells. It can interact with hyaluronan, collagen, flaminin, fibronectin, and osteopontin. In particular, the osteopontin binding with the CD44 isoforms v6 and v7 was found to promote cell survival, migration, invasion, and angiogenesis [138]. Moreover, CD44 is responsible for the activation of key signaling pathways in both malignant and non-malignant cells, such as Rho GTPases, Ras/MAPK, and PI3K/AKT. When CD44-ligand binding occurs, CD44 causes the formation of clusters in the extracellular matrix with the subsequent activation of kinases, such as T-lymphoma invasion and metastasis-inducing protein 1 (Tiam1), p115, Ras-related C3 botulinum toxin substrate 1 (Rac1), c-Src, Focal adhesion kinase (FAK), Ras homolog (Rho), and Rac, thus promoting cell migration [138].

Finally, Roundabout (Robo) receptors are proteins lacking any catalytic activity, thus depending on other molecules activation. In this case, its activity is strictly related to the Slit proteins, secreted glycoproteins, including Slit1, Slit2, and Slit3. The Slit2/Robo4 signaling regulates cell migration, cell proliferation, and vascular permeability due to high Robo4 expression by endothelial cells. Other molecules regulating Robo functions are Sdf1 and Cxcr4, both playing a pivotal role in hematopoietic stem cell (HSC) location and function [139,140].

### 2.3. Combinatory Stimulation of Topographical and Biochemical Guidance

As mentioned previously, in Section 2.2, the biomechanical contact guidance mechanism has been exploited in tissue engineering for the development of bioactive biomaterials influencing cells behavior by their specific topography. In fact, different cell types preferentially adhere to specific topographies, so, by modifying surface properties, it is possible to directly influence cell behavior. One clear example is given by fibroblasts, cells representative for the soft tissues. Even if they are strictly rugophobic, Ferraris et al. [141] demonstrated that nanostructured titanium alloys are suitable platform for cell adhesion, spreading and orientation. Similarly, other recent studies [142,143,144] suggested that nanotopographies, including nanofibers, nanogrooves, nanocolumns, nanopillars, nanotips, and dots, are able to influence cell behavior increasing cell adhesion, differentiation, and spread, too. Moreover, in order to maximize the contact guidance effect, it is important to precisely consider the topography of the surface elements, as well as their distance. In literature, some examples are given involving fibroblasts, endothelial cells, osteoblasts, and keratinocytes.

To orient fibroblast using nanofibers and nanogrooves, fixed measured have been reviewed by Spriano et al. [145]; accordingly, the grooves must be deeper than 35 nm in order to be bigger than the collagen fibrils and preferentially large, around 20 microns, in order to allow cells to reach the surface of the grove. Moreover, fibers and grooves height must be lower than 2 microns in order to allow easy interconnections among cells adherent on different grooves [146]. Endothelial cells alignment can be reached when type I collagen nanofibers are between 30 and 50 nm, when micropatterns of fibronectin stripes are between 2.5 and 100 microns, or when microgrooves height range between 200 nm and 10 microns and depth is between 50 nm and 5 microns [146]. Osteoblasts alignment is reached when nanogrooves have 300 nm frequency and 60–70 nm depth. Acceptable keratinocytes alignment is reached when grooves hold at least 800 nm height; in fact, lower ridges induce consequentially a reduction in cell alignment. In the case of keratinocytes, if alignment is reached only with grooves, adhesion is higher in smooth surface [146].

Interesting, topography does not modify only cells’ shape, but it can also influence their phenotype or even genotype. In fact, when cytoskeleton microfilaments, microtubules, and intermediate filaments adapt to the substrate nanotopography, the laminae and the nucleus are also affected by such rearrangement, thus regulating gene expression. For example, Werner et al. [147] demonstrated that convex surfaces, typically present in nanofibers, induced nuclear compression of mesenchymal stem cells, thus leading to a high lamin-A expression level and, consequently, to osteogenic differentiation. On the opposite side, Wise et al. [148] demonstrated that a scaffold mainly composed of nanofibers (having convex surface) influenced mesenchymal stem cell differentiation towards chondrogenic-like phenotype. Those contrasting results evidence how the size and distance among fibers can influence gene expression and cells maturation. Another example of the nuclear compression effect on differentiation is given by Pan et al. [149], who demonstrated that nano-topographic-induced nuclei compression in induced pluripotent stem cell (iPS) can lead to neuronal markers expression and consequently to neuronal lineage differentiation or to hepatic differentiation according to the surface’s topographic arrangement.

### 2.4. Biological Advantages of Cytocompatible Nanofibers-Based Biomaterials

As previously debated, nanotopographies are suitable tools to influence cells behavior, thus potentially playing and important role in stimulating cells repopulation of implantable materials aimed at injured tissue healing. Tissues in the human body are mainly divided into hard and soft tissues: hard tissues include bone and teeth, while soft tissues are connective and supporting surrounding structures and organs of the body, including ligaments, tendons, and muscles. Hard tissues are usually replaced by titanium and its alloys (e.g., Ti6Al4V), while soft tissues are by polymers or composites [150,151]. All the nanotechnologies previously mentioned (e.g., nanogrooves, nanocolumns, nanofibers, nanopillars, nanopits) can be exploited as surface treatments to improve the bioactivity of both hard- or soft-tissue dedicated biomaterials. Among them, nanofibers represent one of the most versatile tools due to the possibility to easily modify their size and orientation by common techniques, such as electrospinning. In fact, they hold a high surface-volume ratio, helping the drug and growth factor delivery; moreover, they are also very similar to natural ECM nanofibrous collagen. Nanofibers can be realized using natural materials (collagen, silk, alginate, chitosan, and hyaluronic acid) [52] or synthetic materials (polycaprolactone (PCL), polyurethane (PU), poly-L-lactic acid (PLLA), polyglycolic acid (PGA)) [52], and copolymer of the two previous substances (such as PLGA) [56]. This large possibility of materials handling makes nanofibers applicable for most tissues, both hard and soft. PLGA electrospun nanofibers were used by Sahoo et al. [152] to reinforce the structure of a knitted device, increasing, at the same time, ECM deposition and surface area. Moreover, the PLGA nanofibers were successfully used as delivery system for biochemical factors (such as FGF) to better control mesenchymal stem cell behavior and its differentiation after mechanical loading [143]. Driscoll et al. [153] reproduced a meniscus construct made by non-aligned or aligned PCL nanofibers, discovering that aligned fibers were 3 times stiffer than non-aligned ones and that they promoted mesenchymal stem cell proliferation and maintenance with a higher matrix deposition. The same PCL nanofibers were used by Shin et al. [154] for the development of a bioactive bone-substitute. They seeded rat mesenchymal stem cell onto PCL nanofibers observing a significant increase of collagen type I synthesis, the major component of bone ECM. Li et al. [155] applied PCL nanofibers for articular cartilage repair. They seeded fetal bovine chondrocytes to evaluate nanofibers sustainment, having as a result a round chondrocytes-like phenotype maintenance, as well as chondrogenic genes up-regulation (aggrecan and collagen type II). These promising results were obtained thanks to the nanofibers ability to form an oriented 3D environment strictly mimicking the complex cartilage geometry that requires specific orientation to support the formation of a collagen II-rich ECM, avoiding the rise of fibrocartilage. Finally, skin is another tissue that can be resembled by nanofibers architecture. In fact, as previously reviewed by Vasita and Katti [156], epidermidis hold low regeneration capability; so, when large skin areas undergo self-healing, the newly formed tissue often lacks proper elasticity and mechanical strength. So, silk fibroin nanofibers were used to produce skin-substitute holding features similar to the naïve tissue; accordingly, Min et al. [157] demonstrated that silk nanofibers allowed for keratinocytes and fibroblast adhesion and spread, thus representing a suitable temporary substrate to support tissue healing, providing the required mechanical properties.

Surface charge of fibers has interesting biological effects. Enhanced focal adhesion formation and osteogenic differentiation of mesenchymal cells have been evidenced on positively charged protein fibers, in a pure protein-based scaffold [158]. Analogously, electrostatic accumulation of Ca^2+^ ions on negatively charged surfaces promotes hydroxyapatite nucleation, which is crucial for further tissue formation and bone regeneration [158]. Surface charge also affects amount and refolding degree of the adsorbed proteins and, thus, cell adhesion and integrin bonding [159]. Surface charge of the biomaterials, especially the electrospun fibers, is most often tuned by surface chemical modifications [160]. Surface charge can be also exploited as a tool to selectively target tumoral cells due to their net negative charge; in fact, while physiological cells possess a neutral or slightly positive charge, tumoral cells possess a marked negative surface charge due to their metabolism, a phenomenon known as the Warburg effect [161]. In fact, tumoral cells’ high glycolysis rate leads to an increase in the lactate acid production and a massive anions release; so, the cations removing results in a negative charge of the surface [162].

Apart from the intrinsic properties of electrospun fibers, great advantages come also from drug uptake and delivery by fiber scaffolds as it is well described in Reference [163,164,165,166,167,168]. Electrospun fibers can be used for drug delivery for oral, trans-dermal, or implantable delivery systems for therapeutic, diagnostic, protection, and filtration purposes or for mimicking the tumor environment [165]. Electrospun fibers with an amorphous structure can maintain an incorporated active ingredient for prolonged periods of time, and they can be utilized to generate oral dosage forms for poorly water-soluble drugs [166]. Concerning tissue engineering and integration, electrospun fibers provide scaffolds with a high degree of porosity like the natural extracellular matrix and large surface area accessible for cell attachment and infiltration, gaseous exchange, mass transfer, and nutrient mobilization. An interesting approach is uptake of active phytoconstituents [167] (e.g., crude extracts, essential oils, phytocompounds) for bone tissue regeneration, cartilage regeneration, skin tissue engineering, antimicrobial dressing, and nerve repair. A great variety of drugs and biomolecules can be uploaded, such as antibiotics, anticancer or chemotherapy, proteins, DNA, RNA, growth factors, and suspensions containing living cells [164].

Finally, dealing with implantable materials it should be taken into account to perform adequate evaluations and safety procedures to test any eventual toxic effect due to the fibers themselves or to the production route. In fact, despite plenty of literature showed nanofibers biocompatibility and high safety levels in vitro, Pogorielov et al. in an in vivo study demonstrated a moderate toxicity following polycaprolactone (PCL) and poly(lactic acid) (PLA) nanofibrous scaffolds subcutaneous, intramuscular and intraperitoneal implantation into rats [169]. In particular, they compared conventional method of electrospinning and NanoMatrix3D technology. In the first case, they noticed rat death in 33% of cases (12 out of 36) 4 weeks after the intramuscular implantation of PCL and PLA. They evidenced hydropic dystrophy, edema, hemorrhages, vascular congestion, and inflammatory infiltrates in kidney and liver. Toxicology exams revealed high concentration of chloroform (often used in the PCL fiber production) in these organs. Results were comparable with the in vitro degradation rate of the fibers showing significative mass loss within 4 to 5 weeks. On the opposite side, no significant side effects and rat death % were noticed with the NanoMatrix3D technology, with kidneys and liver markers in the physiological range. These results do not evidence PCL and PLA nanofibers toxicity (globally recognized as biocompatible) but evidence how conventional electrospinning devices and unsuccessful air conditioning of fibers did not provide the necessary solvent evaporation compared to certified electrospinning devices.

## 3. Topographical and Biochemical Guidance: A Focus on Electrospun Fibers in Implantable Devices

Electrospinning is widely investigated for production of fibrous constructs for several applications in the tissue engineering field, such as skin, muscle, vascular, neural, bone, cartilage, and tendon/ligament soft tissue regeneration, as reported in some recent reviews [47,170,171] and discussed in the previous section of the present review. The main advantages of this technique are versatility in terms of fiber diameter (from nanometers to microns), materials, fiber orientation, and density, as well as reasonable costs and good upscaling ability. On the other hand, the main difficulties in the obtainment of constructs with optimal tissue regeneration ability are insufficient cell infiltration, residual toxic solvents, and poor mechanical properties, as evidenced in Reference [172], with some possible solutions to overcome these issues. The optimization of electrospun nanofibers properties by means of artificial neural networks has also been proposed [173]. Moreover, it has been underlined that, despite numerous successful applications at the laboratory scale, few solutions reached clinical application [171].

The majority of the research works and reviews are focused on the production of scaffold and membranes for tissue regeneration. The present section is focused on the investigation of combination of topographical and biochemical stimuli in electrospun fibers in different constructs (including scaffolds and membranes but also coatings and complex structures) and on the analysis of the role of the different cues in the development of multifunctional implantable devices.

A summary of the considered research studies and their main features is reported in Table 2. The table compares the reviewed research works considering the materials used for fiber production, the produced construct, the possible fiber alignment, the eventual surface functionalization/modification, the target tissue, and the main evidences.

The use of electrospun fibers has been proposed for regeneration of several tissues, among which the most investigated ones are neural, bone, cardiovascular and soft tissues (ligament, muscle). Fiber alignment is crucial for the tissues in which cells are physiologically aligned as neural tissues [174,175,176,177,178,179,180,181,182], blood vessels [183,184], and certain soft tissues, such as ligament, gum, and muscle [41,185,186,187,188,189,190]. In these cases, fibers can guide cells through physiological alignment and growth by means of a contact guidance phenomenon. It has been observed that surface topography (due to fiber orientation or substrate pattern) affects cell orientation and morphology but not viability [191]. Moreover, cells orientation stimulates their maturation state (e.g., Swann cells [182]) or differentiation to ligament/tendon [186]. On the other hand, random fibers can promote osteoblast differentiation [186] and are usually applied for bone regeneration [192,193,194].

The fiber diameter is crucial to obtain cell orientation; in fact, a higher cellular alignment has been observed onto micro-fibers with respect to nanofibers [183,188], due to higher cell interaction with a single fiber in the case of higher diameter with respect to smaller one (multiple contact with fibers aligned at the micro scale but not perfectly at the nano one) [188]. Alignment on nanofibers can be obtained by coupling the mechanical effect with electrical one (chain and molecular orientation) [188]. Moreover, a higher cellular differentiation has been observed for neural stem cells onto polylactic acid nanofibers (300 nm) compared to microfibers of the same material [177]. The effect of fiber diameter on neural cells has also been observed on micro and nano gelatin fibers, evidencing the ability of micro fibers to promote Swann cells migration and axonal outgrowth, while nanofibers can better support the adhesion and proliferation of the same cells [195]. The work underlines the importance of the complex constructs with different fibers diameter for a physiological nerve regeneration.

The possibility to combine different stimuli in fibers (e.g., topographical, electrical, chemical, and biological) has been explored due to the versatility of the electrospinning technique.

Conductive coatings (polypyrrole (PPY)) have been deposited onto electrospun fibers for neural application to impart to the neural cells also an electrical stimulus, typical of the neural tissue [143,148,150]. Moreover, fiber polarization or fiber conductivity (Polyanaline in blend) has been considered in muscle regeneration [188,189].

Doping with specific ions directly on the fibers, such as for Ag^+^ ions onto keratin fibers [97] or by fibers enrichment with bioactive glass nanoparticles doped with cobalt and boron ions [196], has been proposed to confer antibacterial or angiogenic properties, respectively.

Finally, fiber doping with specific molecules has been reported with the aim to impart specific biochemical stimuli to different types of cells. Bone Morphogenetic Protein 2 (BMP-2) [192] and a bone forming peptide [191] have been used for the functionalization of cellulose and Poly(lactide-co-glycolic acid) electrospun fibers for bone regeneration, while heparin [197] has been loaded onto Poly(L-lactide-co-caprolactone) fibers for cardiovascular application (stents for aneurysm treatment). Moreover, curcumin loading in nanopores of Polyvinyl formal (PVF) fibers [198] and ceria nanoparticles into gelatin fibers [199] have been proposed in neural regeneration to impart antioxidant properties.

Most of the works (Table 2) describe fiber mats or scaffolds designed to study the effect of fibers orientation, size, chemical composition, stiffness, and functionalization on stimulation of a specific cell line or tissue. Moreover, some works simulate situations closest to the final clinical application; examples are Poly(lactide-co-glycolide) (PLGA) fibers in conduits for nerve regeneration [176], Poly(lactide-co-glycolic acid) PLGA porous membranes for Guided Bone Regeneration, polycaprolactone complex constructs with bone/ligament compartments with different fiber alignment [187], Poly(L-lactide-co-caprolactone) nanofibers coating on metallic stent for aneurysm treatment, and keratin submicrometric fibers random/aligned on grooved titanium substrates to simulate transmucosal dental implant collars [37,41,97].

The suitability of electrospun fibers aimed at biomedical applications is today demonstrated by the presence of some clinical trials involving the use of such materials. Khan et al., [200] were able to produce chitosan (CH)/poly (ε-caprolactone) (PCL) mucoadhesive hybrid nanofiber membrane functionalized with tinidazole to counteract infections causing periodontal diseases. Functionalized nanofibers were successful in maintaining their antibacterial activity during the 8-weeks clinical trial time, thus significantly improving the patient’s clinical parameters (pocket depth, gingival index, clinical attachment level, and bleeding). Moreover, nanofiber membranes didn’t show any evidence of irritation at the implant site, thus suggesting for high biocompatibility. Other clinical trials were addressed to the use of nanofibers for the chronic wound treatment [201,202,203]. In one of these studies, Arenbergerova et al. [201]. applied antibacterial polyurethane nanofibers textile (NT) doped with the photosensitizer molecule tetra-phenylporphyrin (TPP) to replace bandages for ulcers, inhibiting pathogen proliferation, while contrasting the proliferation of collagen and the re-colonization of granulation tissue by keratinocytes. Results evidenced how after 42 days trial, illuminated TPP-doped-NT reduced the wound-related pain, wound size, and slough tissue and fibrin volume without serious side effects. Moreover, an increase in the healthy granulation tissue and epithelization has been noticed in patients’ carrying nanomaterials. Therefore, some clinical evidences supporting the use of electrospun nanofibers for clinical applications are already available.

Usually, fiber alignment is obtained by mechanical ways (e.g., rotating collectors; see Section 1 of the present review). However, it has been reported that PLA fibers stretching in ethanol solution can result in fiber orientation, with consequent contact guidance effect, without crystallization [199].

## 4. Conclusions

Nano and micro electrospun fibers of various natural and synthetic polymers have been investigated for the regeneration of numerous biological tissues.

An effective role of fiber alignment has been highlighted in the orientation and physiological regeneration of oriented biological tissues (e.g., neural, ligament, and muscle, to cite some examples), as well as in the cells’ fate decision, through the integrins pathways directly influencing the nucleus and the genotype. Conversely, fibers topography seems to lack of an active role towards cells metabolism and cell viability is generally independent on fiber alignment. The size of fiber is another crucial parameter influencing cell behavior and to promote a physiological regeneration. Therefore, current literature demonstrated that electrospun fibers can guide cells’ growth by means of contact guidance phenomena, as well as through physiological alignment. In fact, surface topography can affect cells’ orientation and morphology and, in some cases, can stimulate the maturation state. On the other hand, random fibers can promote cells’ differentiation. Some successfully clinical trials are already available supporting the use of nanofibers-based materials for the treatment of severe diseases, such as chronic wounds.

Finally, topographical stimuli can be successfully combined with the biochemical/biomechanical ones, proper fiber composition, and eventual surface functionalization with ions or molecules. The scientific literature reports numerous promising works in this field; however, few of them consider structures closed to the final medical devices, and clinical applications are up to now mainly related to wound dressings. In this context, new research focused on upscaling of the model samples to real implantable devices are of relevant interest in numerous fields.

## Figures and Tables

**Table 1:** Integrins subunits and their specific extracellular matrix (ECM) proteins substrate.

Subunit(s) α	Subunit(s) β	ECM Target Substrates
α_1_; α_2_; α_3_; α_4_; α_5_; α_6_; α_7_; α_8_; α_v_	β_1_	Collagen; laminin; fibronectin; VCAM-1; RGD
α_L_, α_M_, α_X_	β_2_	ICAM-1,2; C3b complement; fibrinogen; factor X; ICAM-1
α_IIb_, α_V_	β_3_	Fibrinogen, fibronectin, vWF, vitronectin, thrombospondin; osteopontin; collagen IV
α_6_	β_4_	Laminin
α_V_	β_5_	Vitronectin
α_V_	β_6_	Fibronectin, TGF- β1
α_4_, α_IEL_	β_7_	V25, VCAM-1
α_V_	β_8_	TGF- β1

**Table 2:** Topographical and biochemical cues in electrospun fibers for various implantable device applications (in vitro/in vivo studies).

Material	Construct Type	Fiber Orientation	Additional Cue	Application	Main Results	Ref.
Polystyrene (PS)	Sub-micron fibers (0.7–0.9 µm diameter)	Different degree of alignment (from random to 95% alignment)	-	Neural regeneration	Human astrocytoma epithelial-like cells exhibited elongated and aligned morphology (suitable for nerve regeneration) on aligned fibers.	[174]
Poly-Methyl-Methacrylate (PMMA)	Nanofibers (0.5 µm diameter)	Aligned and random	-	Neural regeneration	Astrocytes aligned and proliferated in suitable way for neural regeneration on aligned fibers.	[175]
Poly(lactide-co-glycolide) (PLGA)	Sub-micron fibers (0.7–0.9 µm diameter) in conduits of the same material	Aligned fibers	Conductive coating (polypyrrole (PPY))	Peripheral nerve regeneration	In vitro promotion of growth and differentiation of neural cells, in vivo (rat model) promotion of sciatic nerve regeneration.	[176]
Polyvinyl formal (PVF)	Scaffold of submicrometric fibers	Random	Nanoporous surface Optional curcumin loading	Neural regeneration	Good adhesion, growth, extension, and viability of hippocampal neurons.	[198]
Poly(L-lactic acid) (PLLA)	Nano (300 nm fiber diameter)/micro (1.5 µm fiber diameter) fibrous scaffold	Random or aligned	-	Neural regeneration	Fiber alignment affects neural stem cells alignment, elongation, and neurite outgrowth, while fiber diameter affects cell differentiation (higher on nanofibers, despite orientation).	[177]
Poly(L-lactic acid) (PLLA)	Porous fibers (0.8 µm fiber diameter, with 200 nm pores)	Random or aligned	Conductive coating (polypyrrole (PPY)). Final fiber diameter 1.24 µm with 100 nm pores)	Neural regeneration (applicable also to other tissues)	Alignment of fibroblasts and neurites increases on aligned, porous, and conductive fibers.	[178]
Gelatin	Nanofibers (200–300 nm diameter)	Random or aligned	Ceria nanoparticles doping	Neural regeneration	Aligned fibers promote neuronal alignment. Ceria nanoparticles improved mechanical properties and introduce antioxidant ability.	[179]
Chitosan (75–85% deacetylated) crosslinked with genipin	Nanofibers (200–300 nm)	Aligned	-	Neural regeneration	Crosslinking effectively limit swelling and degradation. Aligned fibers promote neuronal cells growth and alignment.	[180]
poly(lactic-co-glycolic acid) (PLGA)	Nanofibers (250–360 nm diameter)	Random or aligned	Conductive coating (polypyrrole (PPY))	Neural regeneration	Electrical stimulation increases neurite outgrowth on all fibers, the same stimulation on aligned fibers also increases neurite length.	[181]
Poly(-caprolactone) (PCL)	Scaffold of micrometric (1–2 µm diameter)	Random or aligned	-	Neural regeneration	Aligned fibers promote cellular alignment and more pronounced maturation on human Swann cells.	[182]
Gelatin crosslinked with γ-glycidoxypropyltrimethoxysilane (GPTMS)	Nano (300–600 nm diameter) or micro (1–1.3 µm diameter) fibers	Random	-	Neural regeneration	Micro-fibers promote Swann cells migration and axonal outgrowth, while nanofibers promote Swann cells adhesion and proliferation. The combination of both sizes allows proper nerve regeneration.	[195]
Cellulose with cellulose nanocrystals	Nanofibers scaffolds (300 nm fiber diameter)	Random or aligned	Fiber doping with recombinant human Bone Morphogenetic Protein 2 (rh-BMP2)	Bone regeneration	BMP-2 doping increases ALP production, calcium content and alizarin red in bone marrow stromal cells with no dependence on fiber orientation. On aligned fibers, cell growth and calcium deposition follow fiber direction. Bone formation in vivo (rabbit model).	[192]
PLLA, PDLLA, PEG-PDLLA, PEG-PLLA	Fibers with diameter ranging 0.14–2.1 µm on glass substrate spin coated with the fiber polymer	Random or aligned	-	Bone regeneration	In presence of osteogenic factors in osteoprogenitor cells culture, an increase in cellular spreading and filopodia extension is obtained on fibers of higher diameter.	[193]
Poly(lactide-co-glycolic acid) (PLGA)	Porous membranes (fibers diameter 1 µm)	Random	Dopamine mediated functionalization with bone forming peptide derived from BMP-7	Guided bone regeneration	Increase in ALP production and Ca deposition for bone marrow stromal cells. Increase in bone growth in vivo (mouse model).	[194]
Poly(D-L-lactic-co-glycolic acid) (PLGA)	Mesh of fibers (0.14–3.16 µm diameter)	Different degree of orientation	-	Ligament regeneration	Fibroblasts spreading increased with fibers diameter, spreading, and orientation of fibroblasts increased with alignment.	[185]
Poly(-caprolactone)-poly(ethylene-glycol)	Mats embedded in porous chitosan scaffolds	Random and oriented	-	Periodontal ligament regeneration	Chitosan acts as a glue to keep together fibers layers and induces anti-inflammatory action. Fiber orientation induces tendon/ligament cellular differentiation of bone marrow stromal cells, while random fibers promote osteogenic differentiation. In vivo (rat model) regeneration of periodontal ligament.	[186]
Polycaprolactone	Porous scaffold. Fibers diameter 20 µm Proposal of complex structures with 2 bone compartments and 1 ligament compartment with different fibers alignment.	Aligned fibers	-	Ligament regeneration	Fiber alignment induces alignment in mesenchymal stem cells.	[187]
Poly(caprolactone)	Micro (1.3–2.4 µm diameter) and nano (300–400 nm) fibers	Random and aligned	-	Cardiovascular	Nanofibers enhance endothelial cells adhesion and proliferation, while alignment is enhanced on aligned micro-fibers. Aligned endothelial cells mimic natural vessel orient by blood flow.	[183]
Poly(glycerol sebacate)-poly(caprolactone)	Fibers (1 µm diameter) on patterned substrate (grooves, squares…)	Random	-	Cardiac patch	Surface topography affects cell orientation and morphology, but not viability	[191]
(poly-L-lactide-co-caprolactone)/(poly-L-lactic acid) PLCL/PLLA	Core-shell fibers	Aligned	-	cardiovascular	Endothelial cell adhesion increases with fibers stiffness, however a loss in endothelium integrity is observed (pathological remodeling). Cellular alignment is observed on aligned fibers.	[184]
Poly(L-lactide-co-caprolactone)	Nanofibers on metallic stent	Random	Heparin doping	Aneurysm treatment	Effective aneurysm treatment in rabbit model	[197]
Poly(-caprolactone)-PCL + polyaniline (PANi) blend	Nanofibers (350–500 nm)	Aligned		Muscle regeneration	Fiber alignment and electrical conductivity stimulate myoblasts orientation and differentiation as in physiological muscle	[189]
Poly(L-lactide-r-glycolide) (PLGA)	Mesh of nanofibers (500 nm diameter)	Random and aligned		Muscle regeneration	Myoblasts grow well on all the meshes, but align (as in physiological muscle) only on the oriented ones	[190]
Poly(L-lactide-r-glycolide) (PLGA)	Nanofibers (80–740 nm diameter)	Aligned	Fiber polarization (electrical alignment)	Soft tissue regeneration (fibroblast orientation)	For fiber diameter >300 nm, all fibers promote fibroblasts alignment, when d > 300 nm electrical alignment significantly improves fibroblast alignment (fiber polarization parallel to geometrical alignment, higher directional signal to molecular scale receptor). When d < 100 nm nanoimprinting (perfect alignment at the nanoscale) becomes fundamental.	[186]
PLA	mats	Random	Stretching in ethanol for alignment	Soft tissues (test on keratinocytes)	Ethanol stretching induces alignment and contact guidance on keratinocytes, increases elasticity and molecular orientation without crystallization.	[199]
Polycaprolactone			Doping with bioactive glass nanoparticles doped with B and Co ions	Soft tissues with high vascularization (e.g., wounds)	Release of cobalt and boron increases cellular vascular endothelial growth factor.	[196]
Keratin	Submicrometric fibers on titanium (polished/grooved)	Random		Soft tissue adhesion and regeneration	Keratin fibers promote gingival fibroblast adhesion and proliferation. Random fibers effect is higher than the one of substrate grooves (loosening of alignment).	[37]
Keratin	Submicrometric fibers on grooved titanium substrate	Aligned		Soft tissue adhesion and regeneration	Gingival fibroblasts align on keratin fibers parallel to substrate grooves.	[41]
Keratin	Submicrometric fibers on titanium	Random	Ag ion doping	Soft tissue adhesion and regeneration	Keratin fibers promote gingival fibroblast adhesion and proliferation and significantly reduce S aureus adhesion (dose-dependent manner with respect to Ag content) maintaining biocompatibility.	[97]
